# The prevalence of selected non-communicable disease risk factors among HIV patients on anti-retroviral therapy in Bushbuckridge sub-district, Mpumalanga province

**DOI:** 10.1186/s12889-019-8134-x

**Published:** 2020-02-18

**Authors:** Rudy Londile Mathebula, Eric Maimela, Nthembelihle Samuel Ntuli

**Affiliations:** 1 Faculty of Health Sciences, Department of Public Health, University of Limpopo, Polokwane, South Africa; 2 Department of Health, Tintswalo Hospital, Acornhoek, Bushbuckridge, sub-district Mpumalanga Province South Africa

**Keywords:** HIV, ART, Cardiovascular disease risk factors, Prevalence, Body mass index, Bushbuckridge

## Abstract

**Background:**

The rates of non-communicable diseases (NCD’s) appear to be increasing in human immunodeficiency virus (HIV) infected people as compared to non-HIV infected people and this will have major implications for clinical care. The aim of the current study was to profile selected cardiovascular disease risk factors among HIV patients on anti-retroviral therapy (ART) in Bushbuckridge sub-district.

**Methods:**

The current study followed a quantitative cross-sectional study design using a questionnaire which was adapted from World Health Organization STEPwise approach to Surveillance (WHO STEPS). Participants were HIV infected people on ART and data was entered into a computer software Microsoft excel, then imported to Stata 12 for analysis.

**Discussion:**

The overall prevalence of overweight at the initiation of ART amongst the participants was 18.1% and obesity was 11.5% as compared to the time of the study which was 21.4% overweight and 19.6% obese. The average time of ART initiation to study period was 3.6 years. The study findings revealed a significant difference (*p-value 0.006*) between the baseline and current body mass index at time of study for females. Hypertension was found to be having a significant difference (*p-value 0.026 and 0.038*) between the baseline and current body mass index at time of study for males and females respectively. The overall prevalence of hypertension was found to be 34.6%, overweight was 21.4% obesity was 19.6%.

The overall prevalence of abnormal waist circumference was 31.9% and females had a higher prevalence of 42.5% as compared to 4.4% of males. The overall prevalence of smoking 10.8% and alcohol consumption was 21.7%. Males were 22.5 times more likely to be smokers than females (*p* < 0.001) and older people were found to be 0.3 times less likely to consume alcohol as compared to young people.

**Conclusions:**

The high levels of selected risk factors for NCDs among adults on ART in the current study area suggest an urgent need for health interventions to control risk factors in an era of HIV with an aim of reducing multiple morbidity of chronic diseases. Occurrence of NCDs and their risk factors with an aim to achieve positive effects of the long-term ART.

## Background

Approximately more than 35 million deaths are caused by non-communicable diseases (NCDs) on an annual basis. Morbidity and mortality due to NCDs contribute significant threat globally on health and economy of individuals, societies and health systems [1, 2]. The four main NCDs which are being targeted for control globally are cardiovascular diseases (CVDs), chronic respiratory diseases, cancers and diabetes and the selected NCD risk factors also targeted for control are tobacco use, harmful alcohol use, salt intake, obesity, raised blood pressure, raised blood glucose and diabetes, and physical inactivity [2–4]. CVDs generally refers to conditions that involve narrowed or blocked blood vessels that can lead to a heart attack, chest pain (angina) or stroke [5]. The development of health systems that are receptive, accessible and equipped to deal with the challenge of prevention and treatment of NCDs is a global priority. Achieving this type of health system will aid in the management of people with NCDs and reduce multiple morbidity [6].

The HIV and acquired immunodeficiency syndrome (AIDS) pandemic has significantly contributed to mortality rates in many countries over the past three decades [7, 8]. There are substantial concerted efforts and actions which have been made to control the epidemic [7]. This include amongst others the introduction of effective antiretroviral therapy (ART) which has substantially reduced AIDS-related mortality [9, 10]. However, non-HIV-related mortality, such as that attributable to CVD, has become increasingly important for the millions of people living with HIV (PLHIV) [7]. Due to the successful control of HIV viremia and HIV-induced AIDS through ART, CVD has emerged as a leading cause of death in those infected with HIV [11]. Cardiovascular diseases are a widely recognised as a complication of HIV infection. Most of the traditional risk factors of CVDs present in the general population are also present among the HIV-infected population [12]. Therefore, the use of antiretroviral therapy among HIV infected population has improved the quality and life expectancy [7, 13, 14] however, this exposes them to the effects of aging [15], including influence of environmental risk factors known to act in general population and contributing to the occurrence of obesity, diabetes mellitus and cardiovascular disease [12, 14, 16] and increased non-HIV related mortality estimated at 33.3 million [16].

Several studies in Africa cited a high burden of hypertension, obesity and hypercholesterolaemia among HIV patients on ART [17, 18]. HIV and Antiretroviral therapy (ART) seem to be causally linked with early CVD even after controlling for NCD’s traditional risk factors and age [19]. An insight into the extent of the burden of NCD’s risk factors amongst HIV positive people on ART is crucial for effective advocacy and action. It is vital to have surveillance for NCD risk factors amongst HIV positive population mainly for planning, implementation and evaluation of health programmes using good policies [20]. Therefore, the aim of this study was to determine the profiles of NCD risk factors among HIV positive people on ART in a rural area of Bushbuckridge in Mpumalanga Province of South Africa. Therefore, there were two specific objectives which were firstly to determine the prevalence of NCD risk factors and how they differ from baseline values among PLHIV on ART; and secondly to determine socio-demographic and clinical predictors of NCD risk factors among the study population.

## Methods

The current study followed a quantitative approach which the design was cross-sectional in nature [21], to describe non-communicable disease risk factors among HIV patients on ART in Bushbuckridge Sub-district of Mpumalanga Province. A total of 372 HIV positive people on ART at Rixile ART Clinic which is attached to Tintswalo Hospital were randomly selected to participate in the study. Therefore, a total of 332 people (240 females and 92 males) completed the adapted WHO STEPS questionnaire. The reasons for not participating included amongst others refusals and others terminated the interview before completion.

The adapted STEPS for NCD risk factors [20, 22] was used to collect information on selected behavioural risk factors through face-to-face interviews and physical measurements [20, 22, 23]. The OMRON M6 and M5-I Digital Automatic Blood Pressure Monitors were used to measure resting blood pressure. Blood pressure was measured three times and the average of the last two readings was used [20]. Criteria for the diagnosis of hypertension were those proposed by World Health Organization (WHO)/International Society of Hypertension using the average systolic BP of 140mmHg or higher, or if the average diastolic BP was 90mmHg or higher, or if participants were on anti-hypertensive treatment [20, 24].

Height and weight were measured once using a stadiometer and digital balance respectively. The readings were recorded to the nearest 0.5 centimetre and to the nearest 0.1 kg, respectively. Participants were measured without shoes and wearing only light clothing. Waist circumference was measured once using a constant tension tape and recorded to the nearest 0.1 cm (High Waist Circumference >102 cm for men and >88 cm for women) [24]. It should be noted that for the initial physical measurements, a retrospective data from the clinic was used which was collected when the patients were registered for treatment. As such not all measurements or risk factors were recorded at the initial enrolment on treatment such as waist circumference, smoking and alcohol intake.

### Statistical methods

Data analysis was conducted through the use of STATA statistical software (STATA Corporation, College Station, Texas) version 12. Categorical variables were presented as percentages whilst continuous variable were expressed as mean ± SD. The coding of data was done in line with WHO guidelines [20]. Comparison of categorical variables was performed using Chi-Square and a level of 0.05 was considered significant. We report 95% confidence intervals (95% CIs) on all proportions. Univariate logistic regression was employed to determine predictors of selected cardiovascular disease risk factors [20].

## Results

A total of 332 participants took part in the current study and majority (72%) of the participants were females as compared to males and there was a statistical significance difference (*p-value 0.001*) between the age groups. The overall age distribution among participants increased with increasing age from 4.2% in age group 18–24 years to 30.4% in age group 35–44 years then dropped to 26.5 and 17.5% in age groups 45–54 years and above 55 years respectively. Considering gender distribution, 7.6% of the male participants were in age group 18–24 years; 31.6% in age group 45–54 years then dropped to 27%. Amongst females, a similar trend was witnessed, from 2.9% in age group 18–24 years to 33.8% in age group 25–44 years but then dropped to 24.6 and 13.8% in age groups 45–54 years and above 55 years respectively as presented in Table 1. Approximately 44.6% of males were unemployed as compared to 66.3% of females and approximately 36% of males were employed as compared to 26% of females. Majority of females were single at 45.8% whereas 51.1% of males were married. Lastly majority of the participants had a primary school level of education followed by no formal school as illustrated in Table 1.

**Table 1:** Socio-demographic information of participants

	Males (*n* = 92)	Females (*n* = 240)	*P*-value
	n (%)	n (%)	
18–24	7 (7.6)	7 (2.9)	< 0.001
25–34	11 (12)	60 (25)	
35–44	20 (21.7)	81 (33.8)	
45–54	29 (31.6)	59 (24.6)	
≥55	25 (27)	33 (13.8)	
Employment status			
Employed	33 (35.9)	62 (25.8)	< 0.001
Self- employed Unemployed	15 (16.3)	15 (6.3)	
Student	41 (44.6)	159 (66.3)	
	3 (3.3)	4 (1.7)	
Marital status			
Single	13 (14.2)	110 (45.8)	< 0.001
Married	47 (51.1)	42 (17.5)	
Co-habiting Divorced	19 (20.7)	29 (12.1)	
Separated	4 (4.4)	14 (5.8)	
Widowed	5 (5.4)	15 (6.3)	
	4 (5.4)	30 (12.5)	
Educational status			
No formal school	31 (33.7)	72 (30)	0.855
Primary school Secondary school	41 (44.6)	104 (43.3)	
Tertiary	18 (19.6)	55 (23)	
Post-graduate	2 (2.2)	8 (3.3)	
	0 (0.0)	1 (0.3)	

### Baseline and current body mass index and blood pressure

Comparing the baseline and current body mass index at time of study, overweight at the initiation of ART amongst the participants was 18.1% and obesity was 11.5% as compared to at the time of the study which was 21.4% overweight and 19.6% obese. The current study findings revealed that there is no significant difference for males, however overweight percentage increased from 13 to 20.7%. The baseline and current body mass index at time of study for females showed that there is a significant difference at *p*-value 0.006. Obesity increased from 14.6 to 25.8% in females. The blood pressure among males indicated a significant difference at *p*-value 0.026 and stage 2 hypertension category increased from 0 to 7.6%. The blood pressure among females also indicated a significant difference at *p*-value 0.038 and pre-hypertension increased from 17.1 to 22.5% whereas stage 2 hypertension dropped from 3.3 to 0.8% (Table 2).

**Table 2:** Baseline and current body mass index and blood pressure for males and females separately

	Males (*n* = 92)		*P*-value	Females (*n* = 240)		*P*-value
	Initial	Current		Initial	Current	
BMI	n (%)	n (%)		n (%)	n (%)	0.006
Underweight	11 (12.0)	10 (7.5)		31 (12.9)	18 (7.5)	
Normal	66 (71.7)	60 (65)	0.590	126 (52.5)	108 (45.0)	
Overweight	12 (13.0)	19 (20.7)		48 (20.0)	52 (21.7)	
Obesity	3 (3.3)	3 (3.3)		35 (14.6)	62 (25.8)	
Blood pressure	n (%)	n (%)	*P*-value	n (%)	n (%)	*P*-value
Hypotension	2 (2.2)	0 (0.0)		9 (3.8)	4 (1.67)	0.038
Normal	66 (71.7)	54 (58.7)	0.026	170 (70.8)	159 (66.2)	
Pre-hypertension	18 (19.6)	23 (25)		41 (17.1)	54 (22.5)	
Stage 1 hypertension	6 (6.5)	8 (8.7)		12 (5.0)	21 (8.7)	
Stage 2 hypertension	0 (0.0)	7 (7.6)		8 (3.3)	2 (0.83)	

### Prevalence of selected risk factors

The overall prevalence of hypertension was found to be 34.6% and males had the highest prevalence at approximately 41% as compared to 32% of females. In breaking down hypertension into different stages, the overall prevalence of pre-hypertension was high at 23.2% and males had a prevalence of pre-hypertension of 25% as compared to 22.5% of females. Stage 1 hypertension had an overall prevalence of approximately 8.7% and both males and females had the same prevalence. Stage 2 hypertension had the lowest overall prevalence of 2.7% and males had a prevalence of 7.6 which is 6.8% higher than females (Table 3).

**Table 3:** Overall prevalence of selected risk factors stratified by gender

Risk factor	Overall %(95%CI)	Males % % (95%CI)	Females %(95%CI)	*P*-value
Hypertension (BP > 140/90 mmHg)	34.6 (29.5–39.8)	41.3 (31.2–51.5)	32.1 (26.1–38.0)	0.114
Pre-hypertension	23.2 (18.6–27.8)	25.0 (16.1–33.9)	22.5 (17.2–27.8)	0.629
Stage 1 hypertension	8.7 (5.7–11.8)	8.7 (2.9–14.5)	8.8 (5.2–27.8)	0.987
Stage 2 hypertension	2.7 (0.9–4.5)	7.6 (2.1–13.0)	0.8 (−0.03–2.0)	0.001
Overweight (BMI kgm^2^ ≥ 25 to ≤29.9)	21.4 (17.0–25.8)	20.7 (12.3–29.0)	21.7 (16.4–26.9)	0.840
Obesity (BMI kgm^2^ ≥ 30)	19.6 (15.3–23.9)	3.3 (− 0.04–6.9)	25.8 (20.3–31.4)	< 0.001
Waist circumference	31.9 (26.9–37.0)	4.4 (0.1–8.6)	42.5 (36.2–48.8)	< 0.001
Smoking	10.8 (7.5–14.2)	32.6 (22.9–42.3)	2.5 (0.5–4.5)	< 0.001
Alcohol	21.7 (17.2–26.1)	39.1 (29.1–49.2)	15.0 (10.5–19.5)	< 0.001

The overall prevalence of overweight was 21.4% and females had a slightly higher prevalence than males at 21.7 and 20.7% respectively. Obesity had an overall prevalence of 19.6% and females had a higher prevalence than males with 22.5%. The overall prevalence of abnormal waist circumference was 31.9% and females had a higher prevalence of 42.5% as compared to 4.4% of males. The overall prevalence of smoking 10.8% and males had a higher prevalence at 32.6% as compared to 2.5% of females. Lastly, the overall prevalence of alcohol consumption was 21.7% and similarly to smoking, males had a high prevalence at 39.1% as compared to 15% in females (Table 3).

### The predictors of selected cardiovascular disease risk factors

The findings of the current study show that in univariate logistic regression, older people were 1.6 times more likely to be hypertensive than young ones. Participants who were widowed were 2.1 times more likely hypertensive than participants who were married. Participants who were on ART regimens (Dumiva and efavirenz) and (Dumiva and alluvia) respectively were 2.1 and 3.3 times more likely hypertensive than participants who were on Fixed-Dose Combination (FDC) therapy (*p* < 0.005) (Table 4). In multivariate logistic regression, older participants were 1.9 times more likely to be hypertensive than younger participants and participants who were on ART regimens Dumiva and alluvia respectively were 3.2 times more likely hypertensive than participants who were on FDC (*p* < 0.05) (Table 5).

**Table 4:** Univariate logistic regression to determine predictors of selected cardiovascular disease risk factors

Variables	Smoking	Alcohol consumption	Hypertension	Overweight	Obesity	Waist Circumference
Age						
18–34 years	Reference (1)	Reference (1)	Reference (1)	Reference (1)	Reference (1)	Reference (1)
≥ 35 years	1.5 (0.5–4.8) ^a^	0.3 (0.2–0.6)**	1.6 (1.3–2.1)***	1.5 (0.8–3.1) ^a^	0.7 (0.4–1.4) ^a^	0.97 (0.53–1.78) ^a^
Gender						
Female	Reference (1)	Reference (1)	Reference (1)	Reference (1)	Reference (1)	Reference (1)
Male	18.9 (0.5–47.4)***	3.6 (2.1–6.3)***	1.5 (0.9–2.4) ^a^	0.9 (0.5–1.9) ^a^	0.09 (0.03–0.3)***	0.3 (0.3–0.5)***
Educational status						
High	Reference (1)	Reference (1)	Reference (1)	Reference (1)	Reference (1)	Reference (1)
Low	0.9 (0.6–1.5) ^a^	0.7 (0.5–1.1) ^a^	0.8 (0.6–1.1) ^a^	1.6 (1.2–2.3)**	1.0 (0.6–1.9) ^a^	1.1 (0.8–1.6) ^a^
Marital status						
Married	Reference (1)	Reference (1)	Reference (1)	Reference (1)	Reference (1)	Reference (1)
Single	3.6 (1.3–9.9)^*^	0.9 (0.4–1.9) ^a^	1.7 (0.9–3.0) ^a^	0.8 (0.4–1.7) ^a^	0.7 (0.3–1.5) ^a^	0.8 (0.4–1.6) ^a^
Cohabiting	3.9 (1.3–11.9)^*^	0.9 (0.4–2.1) ^a^	1.1 (0.5–2.4) ^a^	0.3 (0.1–1.0)*	0.7 (0.2–1.9) ^a^	0.7 (0.3–1.9) ^a^
Divorced	5.6 (1.4–22.2)*	0.7 (0.2–3.1) ^a^	2.2 (0.7–6.0) ^a^	0.2 (0.02–1.2) ^a^	3.1 (0.9–10.4) ^a^	1.6 (0.5–5.1) ^a^
Separated	2.2 (0.4–11.6) ^a^	0.2 (0.02–1.4) ^a^	2.2 (0.8–5.9) ^a^	0.8 (0.2–2.6) ^a^	0.6 (0.2–2.6) ^a^	1.1 (0.4–3.7) ^a^
Widowed	1.2 (0.2–6.3) ^a^	0.4 (0.1–1.6) ^a^	2.7 (1.2–6.0)^*^	1.0 (0.4–2.6) ^a^	1.0 (0.3–2.9) ^a^	1.6 (0.6–4.1) ^a^
Work status						
Working	Reference (1)	Reference (1)	Reference (1)	Reference (1)	Reference (1)	Reference (1)
Not working	0.8 (0.5–1.1)^a^	2.8 (0.7–10.8) ^a^	0.9 (0.7–1.1)^a^	0.08 (0.02–0.3)^a^	1.2 (0.3–5.1) ^a^	0.8 (2.1–2.9) ^a^
ART Regimen						
FDC	Reference (1)	Reference (1)	Reference (1)	Reference (1)	Reference (1)	Reference (1)
Dumiva and efavirenz	0.9 (0.3–2.2)^a^	0.3 (0.1–0.7)*	2.1 (1.1–3.8)*	0.8 (0.4–1.7)^a^	1.7 (0.8–3.3)^a^	2.1 (1.2–3.8)*
Dumiva and alluvia	0.6 (0.1–2.7)^a^	0.7 (0.2–1.9)^a^	3.3 (1.4–7.5)**	1.4 (0.5–3.4)^a^	0.8 (0.3–2.6)^a^	1.2 (0.5–2.8)^a^
Efavirenz and lamividine	–	–	–	3.7 (0.2–59.7)^a^	–	2.6 (0.2–42.4)^a^
Tenamine and neverapine	0.4 (0.05–3.2)^a^	0.3 (0.1–1.5)^a^	1.7 (0.7–4.5)^a^	0.7 (0.2–2.5)^a^	1.6 (0.6–4.8)^a^	1.9 (0.7–4.9)^a^
Alluvia and tenefovir	–	1.9 (0.3–11.7)^a^	–	2.4 (0.4–15.0)^a^	1.2 (0.1–10.6)^a^	1.7 (0.3–10.6)^a^
Duration of ART						
≥ 5 years	Reference (1)	Reference (1)	Reference (1)	Reference (1)	Reference (1)	Reference (1)
3–4 years	0.8 (0.3–2.5)^a^	0.6 (0.2–1.6)^a^	1.6 (0.7–3.5)^a^	0.7 (0.3–1.5)^a^	1.6 (0.8–3.2)^a^	0.8 (0.2–4.2)^a^
≤ 2 years	1.2 (0.5–2.9)^a^	1.4 (0.7–3.0)^a^	0.6 (0.3–1.3)^a^	1.7 (0.9–3.0)^a^	0.9 (0.4–1.7)^a^	0.3 (0.04–2.5)^a^

Values are reported as odds ratios (95%CI); * significant at *p* < 0.05; ** significant at *p* < 0.005; *** significant at *p* < 0.001, ^a^Not significant

**Table 5:** Multivariate logistic regression to determine predictors of selected cardiovascular disease risk factors

Variables	Smoking	Alcohol consumption	Hypertension	Overweight	Obesity	Waist Circumference
Age						
18–34 years	Reference (1)	Reference (1)	Reference (1)	Reference (1)	Reference (1)	Reference (1)
≥ 35 years	1.7 (0.5–5.2) ^a^	0.4 (0.2–0.7)***	1.9 (1.1–3.5)*	–	–	–
Gender						
Female	Reference (1)	Reference (1)	Reference (1)	Reference (1)	Reference (1)	Reference (1)
Male	21.9 (7.7–62.1)***	4.9 (2.7–8.9)***	–	–	0.05 (0.02–0.1)***	0.07 (0.02–0.3)***
Educational status						
High	Reference (1)	Reference (1)	Reference (1)	Reference (1)	Reference (1)	Reference (1)
Low	1.9 (0.6–5.8) ^a^	–	–	0.5 (0.3–0.9)*	–	–
Marital status						
Married	Reference (1)	Reference (1)	Reference (1)	Reference (1)	Reference (1)	Reference (1)
Single	0.7 (0.2–2.5)^a^	–	–	1.3 (0.8–2.4) ^a^	1.6 (0.7–3.5) ^a^	–
Cohabiting	1.3 (0.4–4.9)^a^	–	–	0.3 (0.1–1.1)^a^	1.1 (0.4–2.9) ^a^	–
Divorced	4.0 (0.7–21.7)^a^	–	–	0.2 (0.03–1.7) ^a^	4.1 (1.3–12.7)^*^	–
Separated	0.9 (0.1–6.2) ^a^	–	–	0.9 (0.3–3.3) ^a^	0.6 (0.2–2.7) ^a^	–
Widowed	0.8 (0.1–5.3) ^a^	–	–	1.3 (0.5–3.3) ^a^	0.9 (0.4–2.7) ^a^	–
Work status						
Working	Reference (1)	Reference (1)	Reference (1)	Reference (1)	Reference (1)	Reference (1)
Not working	–	–	–	–	–	–
ART Regimen						
FDC	Reference (1)	Reference (1)	Reference (1)	Reference (1)	Reference (1)	Reference (1)
Dumiva and efavirenz	–	0.3 (0.08–0.7)*	1.8 (0.9–3.3)^a^	–	2.4 (1.1–5.5)^*^	3.4 (1.7–7.0)***
Dumiva and alluvia	–	0.7 (0.2–1.9)^a^	3.2 (1.4–7.4)*	–	0.9 (0.3–3.1)^a^	1.2 (0.5–3.1)^a^
Efavirenz and lamividine	–	–	–	–	–	1.7 (0.1–28.4)^a^
Tenamine and neverapine	–	0.4 (0.08–1.8)^a^	1.7 (0.6–4.3)^a^	–	1.8 (0.5–5.6)^a^	1.9 (0.7–5.3)^a^
Alluvia and tenefovir	–	1.5 (0.2–11.0)^a^	–	–	1.9 (0.2–20.4)^a^	2.8 (0.3–24.5)^a^
Duration of ART						
≥ 5 years	Reference (1)	Reference (1)	Reference (1)	Reference (1)	Reference (1)	Reference (1)
3–4 years	–	–	–	0.7 (0.3–1.6)^a^	–	–
≤ 2 years	–	–	–	1.7 (0.9–3.1)^a^	–	–

Values are reported as odds ratios (95%CI); * significant at *p* < 0.05; ** significant at *p* < 0.005; *** significant at *p* < 0.001, ^a^Not significant

In univariate logistic regression, participants with higher educational level were 1.6 times more likely to be overweight than participants with lower educational level (*p* < 0.005) (Table 4) whereas multivariate logistic regression, participants with higher educational level were 0.5 times less likely to be overweight than participants with lower educational level participants (*p* < 0.05) (Table 5). Participants who were males were 0.09 times less likely to be obese than females in univariate logistic regression (*p* < 0.001) (Table 4) whereas in multivariate logistic regression, males were 0.05 times less likely to be obese than females (*p* < 0.001) (Table 5). Participants who were males were 0.3 times less likely to have high waist circumference than females in univariate logistic regression (*p* < 0.001) (Table 4) whereas in multivariate logistic regression, males were 0.07 times less likely to have high waist circumference than females (*p* < 0.001) (Table 5). In univariate logistic regression, participants who were on ART regimens Dumiva and efavirenz were 2.1 times more likely to have high waist circumference than participants who were on FDC (*p* < 0.05) (Table 4) whereas in multivariate logistic regression, participants who were on ART regimens Dumiva and efavirenz were 3.4 times more likely to have high waist circumference than participants who were on FDC (*p* < 0.001) (Table 5).

In univariate logistic regression, older people were 1.5 times more likely to be smokers than younger people but not statistically significant. However, males were 18.9 times more likely to be smokers than females (*p* < 0.001). Participants who were single, cohabiting and divorced were 3.6, 3.9 and 5.6 times more likely to be smokers respectively as compared to participants who were married (*p* < 0.05) (Table 4). In multivariate logistic regression, males were 21.9 times more likely to be smokers as compared to females (*p* < 0.001). With regard to alcohol consumption, older people were found to be 0.3 times less likely to consume alcohol as compared to young people (*p* < 0.005) in univariate logistic regression and in multivariate logistic regression, older people were found to be 0.3 times less likely to consume alcohol as compared to young people (*p* < 0.001). Males were 4 times more likely to consume alcohol as compared to females (*p* < 0.001) and in both univariate and multivariate logistic regression participants who were on ART regimens Dumiva and efavirenz were 0.3 times less likely to consume alcohol than participants who were on FDC (*p* < 0.05) (Table 4 and Table 5). In multivariate logistic regression, males were 4.9 times more likely to consume alcohol than females (*p* < 0.001) than females (Table 5).

## Discussion

The methodology employed in the current study was retrospective quantitative cross-sectional in nature. The study used a questionnaire which was adapted from World Health Organization stepwise approach to surveillance (WHO STEPS) which has been validated and used in several studies globally [25]. Therefore, this signifies reliability of the current study findings.

A cross sectional study conducted in Tanzania revealed that HIV and Non communicable diseases (NCDs) are major problem of public health importance in developing countries [26] which is similar to the current study findings as there is an increase in NCD risk factors amongst HIV positive people.

A number of studies have shown that there may be increased NCD risk factors in HIV-infected versus uninfected populations [27]. An increasing trend in the overall prevalence of overweight at the initiation of ART amongst the participants in the current study was noted which concurs with a study conducted by Tate et al. (2012) in Birminghamin [25]. Remarkably, a greater proportion of females than males were overweight and obese at baseline and during the conduct of the study which is in agreement with the findings from other studies [25, 28]. The highest prevalence of a higher waist circumference (abdominal obesity) (66.7%) is among females in the age category ≥55 years, surprisingly the age category 18 to 24 years among females have a prevalence of 42.9%. The latter, could be due to sedentary lifestyles and dietary patterns that young adults are in engaged in. The current results coincide with a study conducted by Alvarez et al. (2010) in Latin America [29]. Although obesity might have a protective effect on HIV disease progression and AIDS-related deaths; it has harmful health consequences such as CVD [17]. These results show that epidemiologic transition may not only occur in developed countries but also in developing countries. This shows that HIV patients on highly active antiretroviral therapy (HAART) should not be encouraged to consume energy-dense foods higher than the general population as they are also prone to obesity as in the general population.

Hypertension, diabetes, and dyslipidaemia are also more common in HIV infected people [30]. In the current study the overall prevalence of hypertension was 34.6%, this is lower than that found in Cameroon of 38% among HIV patients on HAART [31]. In this study, Men had the highest prevalence of hypertension than women. The prevalence of hypertension among men and women in the current study was 41.3 and 32.1% respectively which concurs with a study conducted in Kenya were males had a higher hypertension prevalence of 11.2% as compared to 7.4% of females [19]. Our study findings again revealed that the highest prevalence of hypertension (60%) was among males in the age category ≥55 years which differs from a study by Bloomfield et al. (2011) where they found younger men had a higher prevalence than older age [19]. The rationale could be that older men may not be able to control their blood pressure and stress levels better than their counterparts of the same age.

This study also found that the overall prehypertension prevalence is 23.2% which was found to be higher than the findings from a study done in three regions of Brazil where it was found that the prevalence of prehypertension was between 3 to 15% [32]. Prehypertension is independently associated with risk of coronary heart disease (CHD), which has been described in subjects not infected with HIV, more studies need to be done in order to find out if prehypertension and HIV are dependently associated with risk of CVD or not. In this study, the chances of having a high prevalence rate of hypertension were predicted by increasing Body Mass Index (BMI). Increasing BMI has been shown to be a predictor of hypertension in a study conducted in Nigeria [28].

Through observation most of the elderly men are staying alone and no one look after them especially after they are diagnosed with HIV. Women also accept conditions or changes in their lifestyle better than men. To support the above statement, blood pressure taken when HAART was initiated and at time of study showed that stage 2 hypertension (≥160/100 mmHg) increased from 0 to 7.6% for men and for women, however decreased from 3.3 to 0.83%. These findings concurred with that of two African studies which showed a higher prevalence of hypertension with HAART [28, 31], thus supporting the fact that HAART could possibly be linked to hypertension in these patients.

The overall smoking rate is 10.8% and according to South African National Health and Nutrition Examination Survey (SANHANES) report of 2013, the prevalence of smoking in South Africa is estimated to be 16%. The highest prevalence of smoking (45.5%) among males in the age category 25 to 34 years and in females highest (12%) in the age category ≥55 years. In our study, the overall alcohol consumption is 21.7% and it is highest (57%) in the age category 18 to 24 years in both males and females. In a study conducted in the United States of America, discovered that the prevalence of alcohol consumption in people living with HIV/AIDS was higher than the general population in the same region [33]. Many studies have found that people with HIV experience age-related comorbid disease, organ system functional decline, and frailty at an earlier age than demographically similar control subjects. This is likely to be accentuated among those consuming harmful amounts of alcohol [34]. Specifically, our study findings revealed that patterns of heavy consumption continue into middle and drops at older ages as compared to other studies [35, 36].

A study conducted in Tanzania Kagaruki et al. (2014) showed that older age (AOR = 3.42, 95% CI 2.06–5.70) was a risk factors that predicted the prevalence of hypertension among participants on ART [28, 37] which concurs with findings from the current study as it was revealed that older age was 1.6 times more likely to be hypertensive (p *< 0.005*). Hypertension was significantly associated with ART regimen Dumiva and alluvia and this concurs with studies conducted in Zimbabwe and Tanzania [28, 38]. Again similar to a study conducted in North-western Tanzania and Southern Uganda [28, 39] there was a significant association between hypertension and age. Male gender has been found to be significantly associated with smoking (AOR = 22.5, 95% CI 7.8–64.7) and alcohol consumption (OR = 4.0, 95% CI 2.1–7.7) in ART patients in the current study which is similar to other studies [35, 36].

## Conclusion

The current study findings support the notion that HIV and NCDs are major problem of public health importance in developing countries. This double burden can be addressed by imposing an integrated approach to management of HIV and NCDs in rural health facilities which will have context-specific factors affecting then integration [40, 41]. The current study then in conclusion, revealed that there is a high level of selected risk factors for NCDs among adults on ART and this suggest an urgent need for health interventions to control risk factors in an era of HIV with an aim of reducing multiple morbidity of chronic diseases. The risk of developing chronic non-communicable diseases is increasingly recognized as a major public health problem in individuals infected with HIV. The profile of patients infected with HIV and on ART is changing and this will have major implications for clinical care in rural areas. The ageing HIV-infected population will put new demands on the health-care systems, which will have important implications for the health of HIV-infected patients in clinical care.

The findings from this study are important to inform health care and training, resource and research priorities and also to establish how non-communicable disease risk factors vary amongst HIV positive population. As Sub-Saharan Africa is facing intersecting epidemics of HIV and hypertension, it is essential to address the occurrence of NCDs and their risk factors with an aim to achieve positive effects of the long-term ART. Together with the adverse effects that HIV and its treatment have on lipids, this may have serious implications for the South African health care system which is burdened by HIV epidemic. Therefore, monitoring of the interaction of HIV, ART use, and non-communicable diseases is needed at both individual and population levels. This can be achieved by strengthening interventions and services which will lead to the integration of HIV/AIDS and NCDs programme or services with other health services and interventions or programs in rural areas.

### Recommendations in light of key study findings

As South Africa and other developing countries have well implemented the roll out of antiretroviral treatment to most people living with HIV and AIDS, it is recommended there be essential data on HIV and NCD comorbidity through a functional surveillance system which will address these conditions simultaneously. Government and politicians should play a key role in supporting the integration of HIV and NCD surveillance systems and in facilitating uptake of this strategy at the country level by encouraging combined surveillance approaches that are tailored to national and local needs.

## Data Availability

The datasets used and/or analysed during the current study are available from the corresponding author on reasonable request.

## Abbreviations

AIDS: Acquired Immunodeficiency Syndrome
ART: Antiretroviral Therapy
BMI: Body Mass Index
CHD: Coronary Heart Disease
CVDs: Cardiovascular Diseases
FDC: Fixed-Dose Combination
HAART: Highly Active Antiretroviral Therapy
HIV: Human Immunodeficiency Virus
NCD’s: Non-Communicable Diseases
PLHIV: People Living with HIV
SANHANES: South African National Health and Nutrition Examination Survey
TREC: Turfloop Research Ethics Committee
WHO STEPS: World Health Organization STEPwise approach to Surveillance
WHO: World Health Organization

## References

[1] Di Cesare M, Khang YH, Asaria P, Blakely T, Cowan MJ, Farzadfar F, Guerrero R, Ikeda N, Kyobutungi C, Msyamboza KP, Oum S (2013). Inequalities in non-communicable diseases and effective responses. The Lancet.

[2] Kontis V, Mathers CD, Rehm J, Stevens GA, Shield KD, Bonita R, Riley LM, Poznyak V, Beaglehole R, Ezzati M (2014). Contribution of six risk factors to achieving the 25× 25 non-communicable disease mortality reduction target: a modelling study. Lancet..

[3] Alleyne G, Binagwaho A, Haines A, Jahan S, Nugent R, Rojhani A, Stuckler D, Lancet NCD (2013). Action Group. Embedding non-communicable diseases in the post-2015 development agenda. The Lancet.

[4] Bonita R, Magnusson R, Bovet P, Zhao D, Malta DC, Geneau R, Suh I, Thankappan KR, McKee M, Hospedales J, De Courten M (2013). Country actions to meet UN commitments on non-communicable diseases: a STEPwise approach. The Lancet.

[5] Martin EA (2015). Concise medical dictionary. Oxford Quick Reference.

[6] Atun R, Jaffar S, Nishtar S, Knaul FM, Barreto ML, Nyirenda M, Banatvala N, Piot P (2013). Improving responsiveness of health systems to non-communicable diseases. The Lancet.

[7] Islam FM, Wu J, Jansson J, Wilson DP (2012). Relative risk of cardiovascular disease among people living with HIV: a systematic review and meta-analysis. HIV Med.

[8] Ortblad Katrina F., Lozano Rafael, Murray Christopher J.L. (2013). The burden of HIV. AIDS.

[9] Eaton JW, Johnson LF, Salomon JA, Bärnighausen T, Bendavid E, Bershteyn A, Bloom DE, Cambiano V, Fraser C, Hontelez JA, Humair S (2012). HIV treatment as prevention: systematic comparison of mathematical models of the potential impact of antiretroviral therapy on HIV incidence in South Africa. PLoS Med.

[10] Pathai S, Bajillan H, Landay AL, High KP (2013). Is HIV a model of accelerated or accentuated aging?. Journals of Gerontology Series A: Biomedical Sciences and Medical Sciences.

[11] Wang T, Yi R, Green LA, Chelvanambi S, Seimetz M, Clauss M (2015). Increased cardiovascular disease risk in the HIV-positive population on ART: potential role of HIV-Nef and Tat. Cardiovasc Pathol.

[12] Nsagha DS, Assob JCN, Njunda AL, Tanue EA, Kibu OD, Ayima CW, Ngowe MN (2015). Risk factors of cardiovascular diseases in HIV/AIDS patients on HAART. The open AIDS journal.

[13] Rooyen JM, Fourie CMT, Steyn HS, Koekemoer G, Huisman HW, Schutte R, Malan L, Glyn M, Smith W, Mels C, Schutte AE (2014). Cardiometabolic markers to identify cardiovascular disease risk in HIV-infected black South Africans. S Afr Med J.

[14] Freiberg MS, Chang CC, Kuller LH, Skanderson M, Lowy E, Kraemer KL, Butt AA, Goetz MB, Leaf D, Oursler KA, Rimland D (2013). HIV infection and the risk of acute myocardial infarction. JAMA Intern Med.

[15] Friis-Møller N, Thiebaut R, Reiss P, Weber R, D'Arminio Monforte A, De Wit S, El-Sadr W, Fontas E, Worm S, Kirk O, Phillips A (2010). Predicting the risk of cardiovascular disease in HIV-infected patients: the data collection on adverse effects of anti-HIV drugs study. Eur J Cardiovasc Prev Rehabil.

[16] Schouten J, Wit FW, Stolte IG, Kootstra NA, van der Valk M, Geerlings SE, Prins M, Reiss P (2014). Cross-sectional comparison of the prevalence of age-associated comorbidities and their risk factors between HIV-infected and uninfected individuals: the AGEhIV cohort study. Clin Infect Dis.

[17] Muhammad S, Sani MU, Okeahialam BN (2013). Cardiovascular disease risk factors among HIV-infected Nigerians receiving highly active antiretroviral therapy. Nigerian medical journal: journal of the Nigeria Medical Association.

[18] Divala OH, Amberbir A, Ismail Z, Beyene T, Garone D, Pfaff C, Singano V, Akello H, Joshua M, Nyirenda MJ, Matengeni A (2016). The burden of hypertension, diabetes mellitus, and cardiovascular risk factors among adult Malawians in HIV care: consequences for integrated services. BMC Public Health.

[19] Deeks SG, Phillips AN (2009). HIV infection, antiretroviral treatment, ageing, and non-AIDS related morbidity. Bmj..

[20] Maimela E, Alberts M, Modjadji SE, Choma SS, Dikotope SA, Ntuli TS, Van Geertruyden JP (2016). The prevalence and determinants of chronic non-communicable disease risk factors amongst adults in the Dikgale health demographic and surveillance system (HDSS) site, Limpopo Province of South Africa. PLoS One.

[21] Parse RR (2003). Research approaches: likenesses and differences. Nurs Sci Q.

[22] Mayega RW, Makumbi F, Rutebemberwa E, Peterson S, Östenson CG, Tomson G, Guwatudde D (2012). Modifiable socio-behavioural factors associated with overweight and hypertension among persons aged 35 to 60 years in eastern Uganda. PLoS One.

[23] Pelzom D, Isaakidis P, Oo MM, Gurung MS, Yangchen P (2017). Alarming prevalence and clustering of modifiable noncommunicable disease risk factors among adults in Bhutan: a nationwide cross-sectional community survey. BMC Public Health.

[24] Mohan V, Deepa M, Farooq S, Datta M, Deepa R (2007). Prevalence, awareness and control of hypertension in Chennai-the Chennai urban rural epidemiology study (CURES–52). J Assoc Physicians India.

[25] World health organization (2015). WHO STEPS instrument: Core and expanded.

[26] Tate T, Willig AL, Willig JH, Raper JL, Moneyham L, Kempf MC, Saag MS, Mugavero MJ (2012). HIV infection and obesity: where did all the wasting go?. Antivir Ther.

[27] Kavishe B, Biraro S, Baisley K, Vanobberghen F, Kapiga S, Munderi P, Smeeth L, Peck R, Mghamba J, Mutungi G, Ikoona E (2015). High prevalence of hypertension and of risk factors for non-communicable diseases (NCDs): a population based cross-sectional survey of NCDS and HIV infection in Northwestern Tanzania and Southern Uganda. BMC Med.

[28] Kagaruki GB, Mayige MT, Ngadaya ES, Kimaro GD, Kalinga AK, Kilale AM, Kahwa AM, Materu GS, Mfinanga SG (2014). Magnitude and risk factors of non-communicable diseases among people living with HIV in Tanzania: a cross sectional study from Mbeya and Dar es Salaam regions. BMC Public Health.

[29] Currier JS, Lundgren JD, Carr A, Klein D, Sabin CA, Sax PE, Schouten JT, Smieja M (2008). Working Group 2. Epidemiological evidence for cardiovascular disease in HIV-infected patients and relationship to highly active antiretroviral therapy. Circulation..

[30] Alvarez C, Salazar R, Galindez J, Rangel F (2010). CastaÃ±eda ML, Lopardo G, Cuhna CA, Roldan Y, Sussman O, Gutierrez G, Cure-Bolt N. Metabolic syndrome in HIV-infected patients receiving antiretroviral therapy in Latin America. Braz J Infect Dis.

[31] Shah K, Alio AP, Hall WJ, Luque AE. The physiological effects of obesity in HIV-infected patient. Journal of AIDS and Clinical Research. 2012;3(4).

[32] Dimala CA, Atashili J, Mbuagbaw JC, Wilfred A, Monekosso GL (2016). Prevalence of hypertension in HIV/AIDS patients on highly active antiretroviral therapy (HAART) compared with HAART-naïve patients at the Limbe Regional Hospital, Cameroon. PloS One.

[33] Bloomfield GS, Khazanie P, Morris A, Rabadán-Diehl C, Benjamin LA, Murdoch D, Radcliff VS, Velazquez EJ, Hicks C (2014). HIV and non-communicable cardiovascular and pulmonary diseases in low-and middle-income countries in the ART era: what we know and best directions for future research. J Acquir Immune Defic Syndr.

[34] Feliciano-Alfonso JE, Mendivil CO, Ariza ID, Pérez CE (2010). Cardiovascular risk factors and metabolic syndrome in a population of young students from the National University of Colombia. Rev Assoc Med Bras.

[35] da Silva CM, Mendoza-Sassi RA, da Mota LD, Nader MM, de Martinez AM (2017). Alcohol use disorders among people living with HIV/AIDS in Southern Brazil: prevalence, risk factors and biological markers outcomes. BMC Infect Dis.

[36] High KP, Brennan-Ing M, Clifford DB, Cohen MH, Currier J, Deeks SG, Deren S, Effros RB, Gebo K, Goronzy JJ, Justice AC (2012). HIV and aging: state of knowledge and areas of critical need for research. A report to the NIH Office of AIDS Research by the HIV and Aging Working Group. J Acquir Immune Defic Syndr.

[37] Braithwaite RS, Conigliaro J, McGinnis KA, Maisto SA, Bryant K, Justice AC (2008). Adjusting alcohol quantity for mean consumption and intoxication threshold improves prediction of nonadherence in HIV patients and HIV-negative controls. Alcohol Clin Exp Res.

[38] Magodoro IM, Esterhuizen TM, Chivese T (2016). A cross-sectional, facility based study of comorbid non-communicable diseases among adults living with HIV infection in Zimbabwe. BMC Res Notes.

[39] Justice A, Sullivan L, Fiellin D (2010). Veterans Aging Cohort Study Project Team. HIV/AIDS, comorbidity, and alcohol: can we make a difference?. Alcohol Res Health.

[40] Nigatu T. Integration of HIV and noncommunicable diseases in health care delivery in low-and middle-income countries. Prev Chronic Dis. 2012;9. 10.5888/pcd9.110331 PMC3431953 22554408

[41] Remais JV, Zeng G, Li G, Tian L, Engelgau MM (2012). Convergence of non-communicable and infectious diseases in low-and middle-income countries. Int J Epidemiol.

